# Supplementary material for: An Update of Bovine Hemoplasmas Based on Phylogenetic and Genomics Analysis
Source: Microorganisms. 2022 Sep 27;10(10):1916. doi: 10.3390/microorganisms10101916 (PMC9607299; doi:10.3390/microorganisms10101916)
Supplement: Supplementary file 1 [file microorganisms-10-01916-s001.zip › microorganisms-1924527-supplementary.pdf]

SUPPLEMENTARY MATERIAL

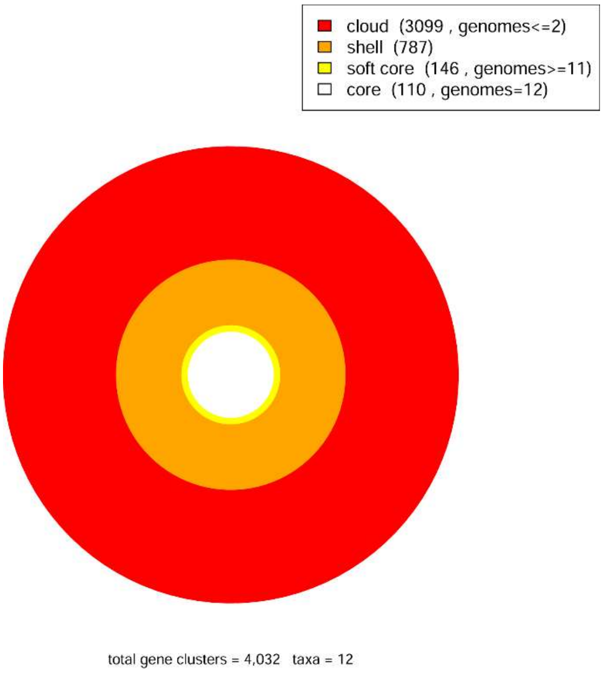

Figure S1. Core, soft core, shell and cloud genomes of hemoplasmas.

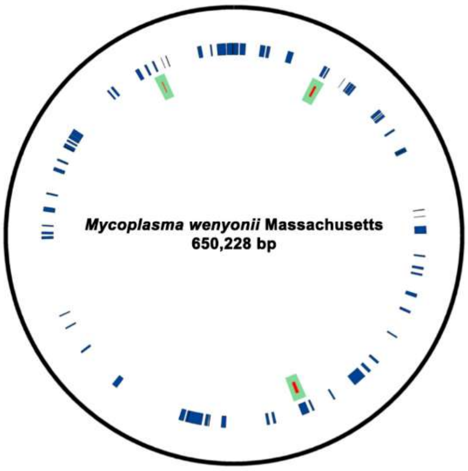

Figure S2. Circular map that compares three bovine hemoplasmas.

Table S1. 12 hemoplasma genomes reported in the GenBank database.

Organism	Host	GenBank
‘*Candidatus* Mycoplasma haemobos’ INIFAP01	Cattle	LWUJ00000000.1
‘*Candidatus* Mycoplasma haemolamae’ Purdue	Camelids	CP003731.1
‘*Candidatus* Mycoplasma haemominutum’ Birmingham 1	Felines	NC_021007.1
Mycoplasma haemocanis Illinois	Dogs	CP003199.1
Mycoplasma haemofelis Langford 1	Cats	FR773153.2
Mycoplasma haemofelis Ohio2	Cats	CP002808.1
Mycoplasma ovis Michigan	Sheep, goats and deer	CP006935.1
Mycoplasma parvum Indiana	Pigs	CP006771.1
Mycoplasma suis Illinois	Pigs	CP002525.1
Mycoplasma suis KI3806	Pigs	FQ790233.1
Mycoplasma wenyonii INIFAP02	Cattle	QKVO00000000.1
Mycoplasma wenyonii Massachusetts	Cattle	CP003703.1

Table S2. BLAST-based Average Nucleotide Identity (ANIb) values of alignment coverage based on the entire genome sequence of 12 hemoplasmas. Coverage values are used to assure minimal coverage requirements for competent matches.

	**'*Ca.* M. Haemobos' INIFAP01**	***Ca.* M. Haemolamae' Purdue**	***Ca.* M. Haemominutum' Birmingham 1**	***M. haemocanis* Illinois**	***M. haemofelis* Langford 1**	***M. haemofelis* Ohio2**	***M. ovis* Michigan**	***M. parvum* Indiana**	***M. suis* Illinois**	***M. suis* KI3806**	***M. wenyonii*INIFAP02**	***M. wenyonii*Massachusetts**
Ca. M. Haemobos' INIFAP01	1	0.0011	0.0011	0.0204	0.0189	0.0203	0.0011	0.0011	0.0029	0.0028	0.0029	0.0021
Ca. M. Haemolamae' Purdue	0.0013	1	0.1500	0.0221	0.0221	0.0248	0.1496	0.1363	0.1480	0.1595	0.1537	0.1534
Ca. M. Haemominutum' Birmingham 1	0.0019	0.2209	1	0.0303	0.0345	0.0262	0.2335	0.2690	0.2298	0.2281	0.2221	0.2169
M. haemocanis Illinois	0.0208	0.0182	0.0169	1	0.7882	0.7761	0.0258	0.0282	0.0233	0.0227	0.0267	0.0264
M. haemofelis Langford 1	0.0154	0.0146	0.0155	0.6320	1	0.9765	0.0159	0.0144	0.0172	0.0164	0.0155	0.0191
M. haemofelis Ohio2	0.0164	0.0162	0.0116	0.6177	0.9692	1	0.0155	0.0164	0.0178	0.0177	0.0152	0.0187
M. ovis Michigan	0.0014	0.1611	0.1708	0.0338	0.0259	0.0255	1	0.2285	0.2081	0.2055	0.4429	0.4337
M. parvum Indiana	0.0018	0.1827	0.2449	0.0459	0.0292	0.0336	0.2844	1	0.5520	0.5496	0.0768	0.2347
M. suis Illinois	0.0036	0.1508	0.1590	0.0288	0.0266	0.0277	0.1969	0.4196	1	0.9088	0.0594	0.1752
M. suis KI3806	0.0037	0.1702	0.1653	0.0294	0.0265	0.0288	0.2036	0.4374	0.9513	1	0.1988	0.1864
M. wenyonii INIFAP02	0.0046	0.1950	0.1913	0.0411	0.0299	0.0295	0.5215	0.0727	0.0739	0.2363	1	0.5158
M. wenyonii Massachusetts	0.0031	0.1786	0.1714	0.0373	0.0337	0.0332	0.4686	0.2037	0.2001	0.2033	0.4733	1

Table S3. BLAST-based average nucleotide identity (ANIb) values of identity based on the entire genome sequence of 12 hemoplasmas. The values of identity of these alignments is declared as the overall genome-to-type-assembly ANI.

	**'*Ca.* M. Haemobos' INIFAP01**	***Ca.* M. Haemolamae' Purdue**	***Ca.* M. Haemominutum' Birmingham 1**	***M. haemocanis* Illinois**	***M. haemofelis* Langford 1**	***M. haemofelis* Ohio2**	***M. ovis* Michigan**	***M. parvum* Indiana**	***M. suis* Illinois**	***M. suis* KI3806**	***M. wenyonii* INIFAP02**	***M. wenyonii* Massachusetts**
Ca. M. Haemobos' INIFAP01	1	0.7134	0.7241	0.7368	0.7377	0.7344	0.7250	0.7336	0.7139	0.7140	0.7412	0.7416
Ca. M. Haemolamae' Purdue	0.7134	1	0.7258	0.7270	0.7325	0.7413	0.7369	0.7215	0.7324	0.7353	0.7354	0.7413
Ca. M. Haemominutum' Birmingham 1	0.7241	0.7258	1	0.7447	0.7277	0.7527	0.7316	0.7376	0.7359	0.7396	0.7334	0.7345
M. haemocanis Illinois	0.7368	0.7270	0.7447	1	0.8247	0.8274	0.7319	0.7343	0.7346	0.7345	0.7291	0.7269
M. haemofelis Langford 1	0.7377	0.7325	0.7277	0.8247	1	0.9741	0.7248	0.7350	0.7357	0.7352	0.7316	0.7297
M. haemofelis Ohio2	0.7344	0.7413	0.7527	0.8274	0.9741	1	0.7493	0.7342	0.7368	0.7358	0.7356	0.7310
M. ovis Michigan	0.7250	0.7369	0.7316	0.7319	0.7248	0.7493	1	0.7452	0.7445	0.7431	0.7826	0.7847
M. parvum Indiana	0.7336	0.7215	0.7376	0.7343	0.7350	0.7342	0.7452	1	0.7784	0.7785	0.7430	0.7414
M. suis Illinois	0.7139	0.7324	0.7359	0.7346	0.7357	0.7368	0.7445	0.7784	1	0.9763	0.7397	0.7500
M. suis KI3806	0.7140	0.7353	0.7396	0.7345	0.7352	0.7358	0.7431	0.7785	0.9763	1	0.7403	0.7501
M. wenyonii INIFAP02	0.7412	0.7354	0.7334	0.7291	0.7316	0.7356	0.7826	0.7430	0.7397	0.7403	1	0.7937
M. wenyonii Massachusetts	0.7416	0.7413	0.7345	0.7269	0.7297	0.7310	0.7847	0.7414	0.7500	0.7501	0.7937	1
